# Characterizing Visual Field Deficits in Cerebral/Cortical Visual Impairment (CVI) Using Combined Diffusion Based Imaging and Functional Retinotopic Mapping: A Case Study

**DOI:** 10.3389/fnsys.2016.00013

**Published:** 2016-02-25

**Authors:** Lotfi B. Merabet, Kathryn J. Devaney, Corinna M. Bauer, Aparna Panja, Gena Heidary, David C. Somers

**Affiliations:** ^1^The Laboratory for Visual Neuroplasticity, Department of Ophthalmology, Massachusetts Eye and Ear Infirmary, Harvard Medical SchoolBoston, MA, USA; ^2^The Attention and Perception Neuroimaging Laboratory, Department of Psychological and Brain Sciences, Boston UniversityBoston, MA, USA; ^3^Pediatric Neuro-Ophthalmology Service, Department of Ophthalmology, Boston Children's Hospital, Harvard Medical SchoolBoston, MA, USA

**Keywords:** cortical/cerebral visual impairment, visual field, perimetry, diffusion MRI, functional MRI, tractography, visual cortex, optic radiations

## Introduction

Cortical/cerebral visual impairment (CVI) is the leading cause of pediatric visual impairment in children in developed countries and has become a significant public health concern (Kong et al., 2012). CVI is clinically defined as significant visual dysfunction resulting primarily from perinatal injury to visual pathways and structures rather than ocular pathology alone (Dutton, 2003). Perinatal hypoxia is the most common cause resulting in impaired maturation of key visual pathways such as the optic radiations; a general condition referred to as white matter damage of immaturity (WMDI). In preterm infants, this maldevelopment is often associated with periventricular leukomalacia (PVL), which is characterized by an enlargement of the lateral ventricles and focal gliosis of surrounding white matter pathways coursing on to the visual cortex (Good et al., 2001; Hoyt, 2007). Depending on the location and extent of the damage, children with CVI often present with a broad range and combination of visual dysfunctions such as decreased visual acuity, visual field deficits, and also impairments in oculomotor, visuomotor, and cognitive visual processing (Good et al., 2001; Dutton, 2003; Hoyt, 2007). The variability in the location and extent of brain injury across individuals makes the prediction of visual functional outcomes and recovery in CVI patients particularly challenging (McKillop and Dutton, 2008).

Despite the increasing prevalence of this condition, the relationship between observed visual deficits in CVI and the underlying structural and functional changes resulting from damage to key visual pathways, remains poorly understood. Specifically, it remains unknown how the maldevelopment of key visual pathways relates to the organization of the visual cortex and further, how these structural and functional changes relate to visual impairments observed within the clinical setting. Standard clinical neuroimaging techniques such as computerized tomography (CT) and magnetic resonance imaging (MRI) can help characterize gross changes in cerebral structure. However, the underlying micro-architecture of key white matter pathways (such as the optic radiations) cannot be fully ascertained, nor can the function of visual cortical areas be assessed. Advances in diffusion based imaging (i.e., diffusion MRI) modalities such as high angular resolution diffusion based imaging (HARDI) combined with tractography analysis techniques can be used to reveal the organization of specific white matter projections (Jones, 2008) see also (Ffytche et al., 2010). At the same time, retinotopic mapping using functional magnetic resonance imaging (fMRI) can be employed to assess the organizational and functional integrity of early visual cortical areas (Wandell, 1999).

In this study, we used a combined structural and functional multi-modal neuroimaging approach to characterize the underlying maldevelopment of the geniculo-striate pathway in an adolescent with CVI. The patient presented here had a documented inferior visual field deficit determined on clinical ophthalmic examination. Despite her diagnosis of CVI and associated visual impairments, she was able to participate in formal testing and provide reliable data (including maintaining fixation during perimetry and retinotopic stimulation) and also remain immobile in the scanner environment without the need of anesthesia. Thus, (and contrary to prior imaging studies with CVI individuals), we had the opportunity to obtain high quality structural and functional imaging data on the same subject in order to investigate the relationship between the structural integrity of the optic radiations and the functional organization of early visual cortical areas with respect to her clinical visual field impairment. We demonstrate the feasibility of combining this structural and functional imaging approach in a patient with CVI along with an age/gender matched normal developed control for comparison. By combining these imaging modalities, it is possible to provide further insight regarding the functional manifestations of early onset developmental damage to key visual pathways and their relation to specific impairments of visual function.

## Case history

The CVI patient (and age/gender matched control subject) and parents provided written informed consent prior to participating in the study. The protocol was approved by the investigative review board of the Massachusetts Eye and Ear Infirmary (Boston, MA, USA) and the study was carried out according to the tenants of the Declaration of Helsinki and conformed to the requirements of the United States Health Insurance Portability and Privacy Act (HIPPA). Ophthalmological examination of the patient was conducted by an experienced pediatric neuro-ophthalmologist. At the time of study, the CVI patient was a 17-year-old girl, born prematurely at 32 weeks gestational age. In the perinatal period, she developed a grade III intra-ventricular hemorrhage with subsequent post-hemorrhagic hydrocephalus, periventricular leukomalacia (PVL), and spastic diplegia. She underwent bilateral strabismus surgery at 11 months to correct an esotropia. Best corrected visual acuity (Snellen) at the time of testing was 20/50 (right eye) and 20/40 (left eye). Her sensorimotor exam was notable for latent nystagmus and a residual microtropia. Funduscopic examination revealed evidence of optic atrophy in each eye, but was otherwise unremarkable. Visual field assessment was performed with automated visual field testing on a Humphrey Field Analyzer using a SITA-Fast 24-2 protocol (Humphrey Field Analyzer 750i, Zeiss Humphrey Systems; Dublin, CA).

As a control, an age and gender matched subject (female, 17 years old) with normal visual acuities and no history of prematurity or neurological/ophthalmic disorders was also recruited for comparison.

## Visual field assessment

**Figure 1:**
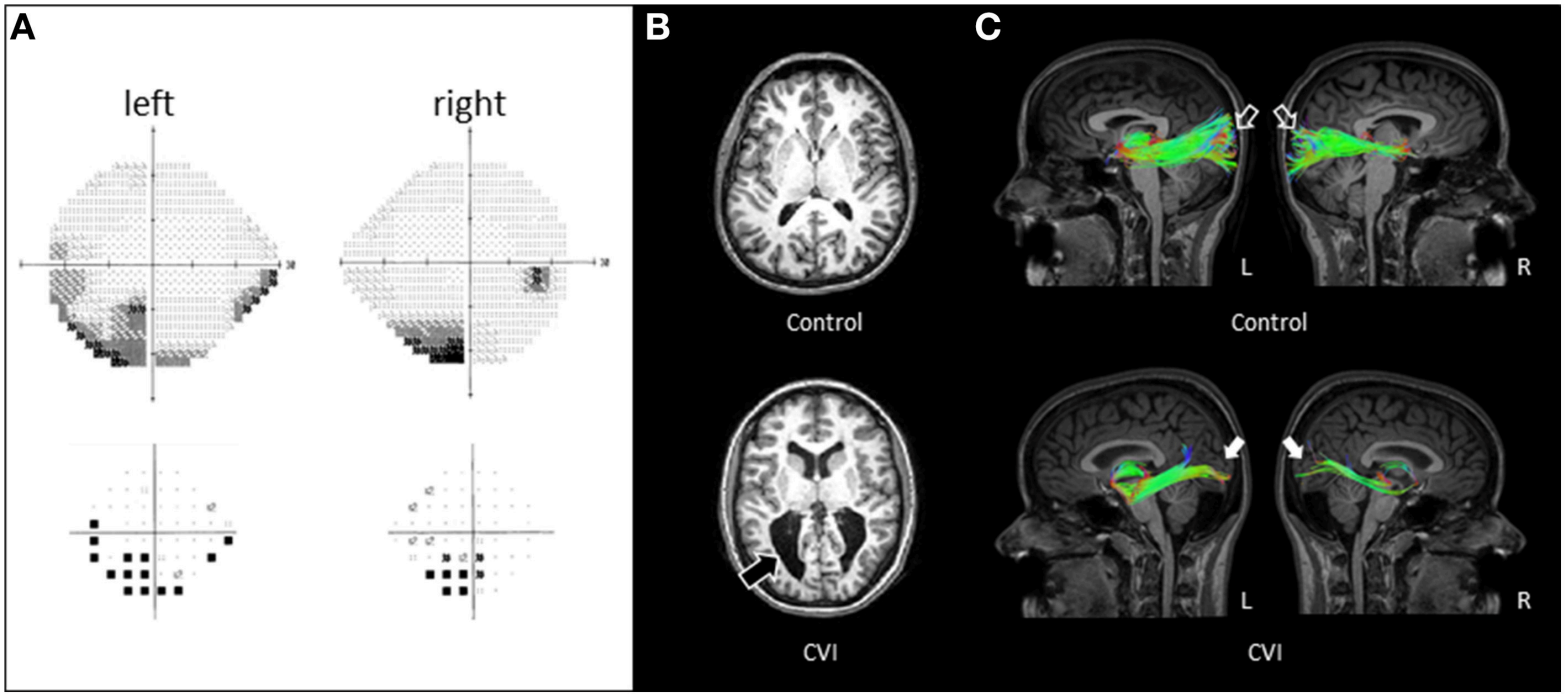
**(A)** Visual field assessment of CVI patient obtained by Humphrey automated visual perimetry (see text for test details). A bilateral field defect involving the inferior visual fields (with a more dense defect involving the left side) in each eye evident on the gray scale plot (top panel) and confirmed on the pattern deviation plot (bottom panel). **(B)** Axial T_1_-weighted MRI images (MP-RAGE pulse sequence) in a normally sighted control (top panel) and CVI patient (lower panel). Enlarged lateral ventricles with irregular posterior borders are apparent in the CVI patient (black arrow). **(C)** Corresponding white matter tractography of the optic radiations (sagittal view) revealed with HARDI in the same individuals. Note in the control subject, the complete arborization of the superior and inferior banks of the optic radiations extending from the thalamus to the occipital cortex in both hemispheres (open arrows). In contrast, the CVI patient shows markedly fewer projections and in particular, along the superior bank of the optic radiations (white arrows). The paucity of connections along the superior bank along with a greater deficit of connections in the right (R) compared to the left (L) hemisphere correspond to the location of the visual field deficit of the CVI patient characterized by automated perimetry.

Automated perimetry using the Humphrey Field Analyzer revealed a stable, bilateral visual field defect involving the inferior visual fields (Figure 1A). Inspection of the pattern deviation plot confirmed the presence of an inferior visual field defect with an apparent greater impairment on the left side. Record review confirmed that the bilateral, inferior visual field loss was non-progressive and identified as early as age 5; when the patient was first able to participate in formal visual field testing.

## Structural and diffusion weighted imaging and white matter tractography

All imaging was carried out using a 3 Tesla Philips Achieva system and an eight-channel head coil. Conventional T1 weighted structural images were acquired using an MP-RAGE pulse sequence (TE 3.1 ms, TR 6.8 ms, flip angle 9 degree, 1 × 1× 1.2 mm voxel size). For white matter tract reconstruction, high angular resolution diffusion imaging (HARDI) was chosen given its superior ability in revealing intravoxel white matter fiber heterogeneity and delineation of multiple fiber orientations within an individual voxel (Tuch et al., 2002). HARDI images were acquired using a single shot EPI sequence (TE 73 ms, TR 17844 ms, 64 directions, Bmax 3000, Bmin 0, 2 mm isotropic voxel size). White matter fiber tracking and reconstruction were performed using DSI Studio software (http://dsi-studio.labsolver.org/) with diffusion decomposition and sparse solution of the fiber orientation distribution function (ODF). HARDI images were aligned to the anatomical data using boundary based registration (Greve and Fischl, 2009). The optic radiations were defined using a two-seed approach. For each hemisphere, the thalamus (composed of the thalamus proper and the ventral diencephalon containing the lateral geniculate nucleus; Desikan et al., 2006) was used as the start seed, while the white matter adjacent to the pericalcarine cortex was used as the second seed. Termination criteria were based on a subject-specific threshold of quantitative anisotropy and an angle change of 75 degree, enabling the capture of the full extent of the optic radiations. Both T1-weighted and HARDI images were acquired in the same scanning session. HARDI images were acquired within a 22 min scan period.

Standard T1-weighted MRI imaging of the CVI patient revealed markedly enlarged ventricles characteristic of PVL as compared to the age/gender matched control (Figure 1B). However, no details regarding the structural integrity of the optic radiations could be ascertained by standard MRI alone. By comparison, white matter reconstruction of the optic radiations obtained by HARDI revealed a generalized reduction in geniculo-striate projections in the CVI patient compared to the normal developed control (sagittal view; Figure 1C). Furthermore, the optic radiations along the superior bank qualitatively appeared to be markedly reduced than in the ventral bank, consistent with the inferior visual field deficit characterized on clinical examination. Finally, the marked reduction of optic radiations in the right hemisphere (compared to left) was also consistent with the laterality of the visual deficit suggested by perimetry findings.

## Functional neuroimaging and visual retinotopy

The functional organization of occipital visual cortex was characterized using static retinotopic mapping techniques allowing for rapid and robust identification of early visual area borders (Rajimehr and Tootell, 2009; Nasr et al., 2011). Boundaries between primary (area V1), secondary (area V2), and tertiary (area V3) visual areas were functionally identified in both hemispheres. Flashing high-contrast colored checkboard stimuli (8 Hz) were presented in sub regions of the visual field and two spatially complementary stimuli were contrasted: (1) a horizontal meridian wedge (8.6° radius and 30° angle) vs. a vertical meridian wedge (8.6° radius and 60° angle) to identify early visual area borders and (2) an upper-field wedge (8.6° radius, 15° angle) vs. a lower-field wedge (8.6° radius, 15° angle) to differentiate activation between the upper and lower visual field. Each pair of stimuli (i.e., meridians as well as upper and lower wedges) were presented in two runs of 16, 16-s blocks comprised of six blocks of each stimulus and four blocks of fixation per run. Retinotopy was acquired with a single-shot EPI sequence (TE 28 ms, TR 2000 ms, flip angle 90 degree, 3 mm isotropic resolution with no slice gap). Each run of the scan was 256 s in duration. For all retinotopic runs, the subjects were instructed to maintain fixation on a central 0.9° fixation stimulus throughout the run and respond using a button box when the central target changed its luminance (TR-wise probability of a luminance change = 30%). Fixation performance (detection accuracy and reaction time) was scored after the scanning session. Stimuli were viewed under binocular viewing conditions.

**Figure 2:**
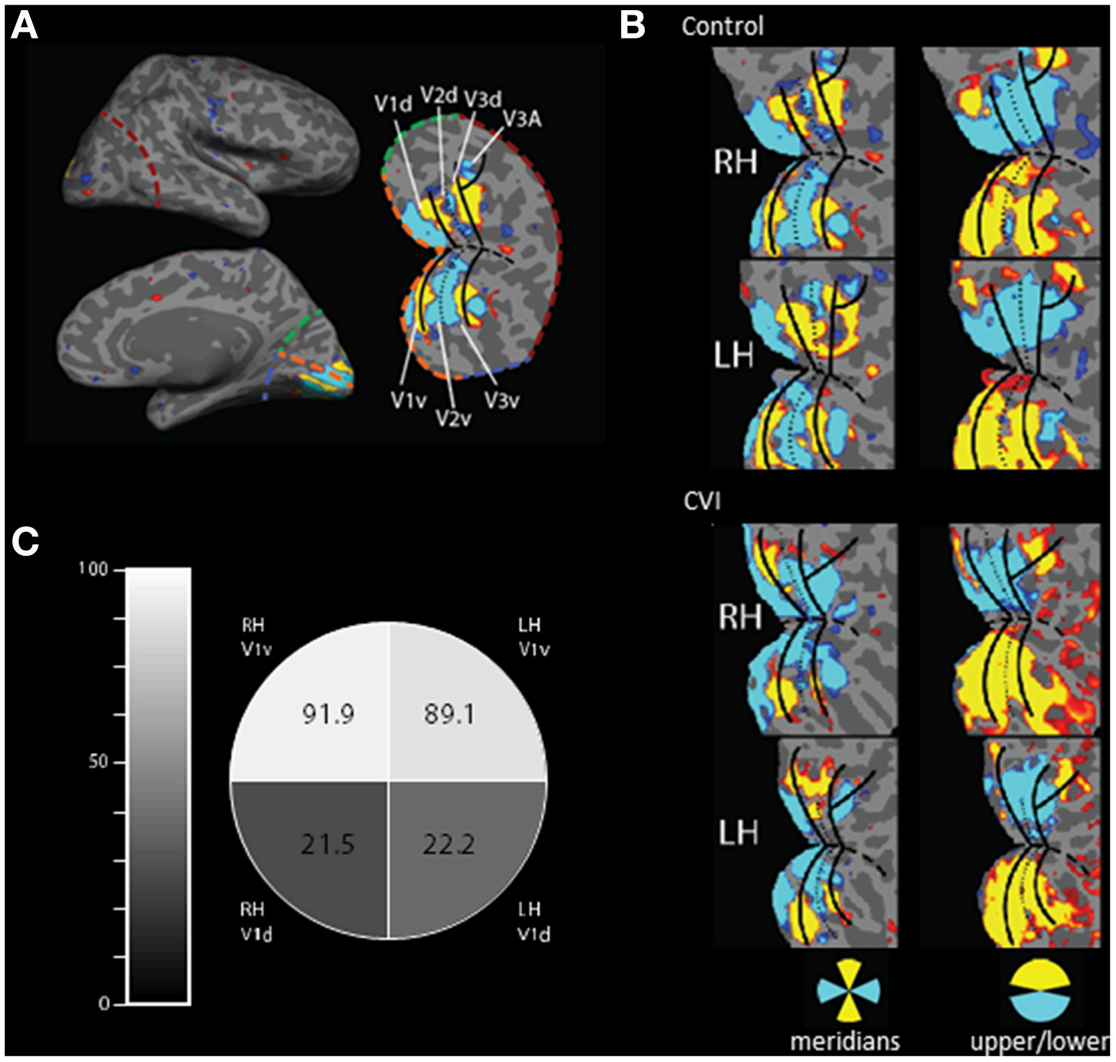
**Activation of early retinotopic areas using fMRI. (A)** Cortical flattening of an occipital cortical patch from the right hemisphere of a normally sighted control. For reference, colored dashed lines are in the same position in both the inflated and flattened views (orange, calcarine sulcus; red, lateral occipital; green, medial, dorsal occipital; and blue, medial ventral occipital). The boundaries of early visual areas (V1–V3) are shown and separated in dorsal (d) and ventral (v) parts. **(B)** Occipital patch projections showing cortical activation in the control (upper panel) and CVI patient (lower panel) in response to visual stimulation using meridians as well as upper and lower wedges (blue and yellow colors correspond to location of activation in response to visual stimulation). Overall, the organization of early visual areas appears largely intact in the CVI patient. However, selectivity for stimulation within the lower visual field was reduced compared to the sighted control (note the left hemisphere is flipped for easier visual comparison). **(C)** Comparison of relative activation in V1 between the CVI patient and the control subject for the cortical representation of each visual field quadrant (expressed as a percentage) confirms that the largest impairment in activation was in dorsal V1 (V1d) and in the right hemisphere (RH) corresponding the left inferior visual field deficit obtained on perimetry.

All structural and functional data were analyzed with FreeSurfer and FS-FAST packages (http://surfer.nmr.mgh.harvard.edu/). Using standard FreeSurfer methods (Fischl et al., 1999; Greve and Fischl, 2009), the surface of each cerebral cortical hemisphere was extracted via image processing based on the anatomical gray-white matter boundary and computationally reconstructed as a 3D mesh of vertices. In order to permit visualization of multiple visual cortical areas, each cortical hemisphere was computationally inflated to reveal buried sulcal regions and an occipital lobe “flat patch” was created for each subject hemisphere by making cuts in the vertex mesh (along the calcarine sulcus and the anterior extent of the occipital lobe) and unfurling this mesh into a 2D representation (see Figure 2A for further details). Each functional run was rigidly registered to the anatomical data using gray-white matter boundary based registration (Greve and Fischl, 2009). Functional data was motion corrected and spatially smoothed using a 3D Gaussian kernel (fwhm = 3.0), separately by run and by hemisphere. Voxel-wise statistical tests were based on a univariate general linear model (GLM), and the significance levels (inverse log *p*-value) were visualized on the inflated and flattened cortical surfaces.

Dorsal (d) and ventral (v) regions of interest (ROI) were identified in each hemisphere and in each participant using block-design retinotopic fMRI analysis (e.g., Nasr et al., 2011). Briefly, the V1/V2 border lies along the center of the vertical meridian response, as it runs both dorsally and ventrally, and the center of the horizontal meridian representation in the calcarine sulcus was used to define dorsal and ventral subdivisions in each cerebral hemisphere. Each ROI represents the diametrically opposed region of the visual field (e.g., dorsal V1 of the right hemisphere represents the lower-left quadrant of the visual field). It is important to note that identifying the boundaries between early visual areas permits us to define ROIs for V1 and V2, but leaves the anterior border of V3 undefined. Since the visual cortical areas just anterior to V3d and V3v (V3A and hV4) contain hemifield representations rather than quadrant representations, the quadrant analysis could be seriously contaminated if the anterior borders are not well localized. For this reason, we quantitatively examined the quadrant results only in areas V1 and V2.

We then determined the BOLD percent signal change to the upper visual field wedge (relative to passive fixation) and to the lower visual field wedge, within each ROI for each subject for each run. Since the dorsal parts of V1 and V2 are responsive to lower visual field stimulation and ventral parts are responsive to the upper visual field stimulation, this allowed us to create an in-field vs. out-of-field metric for each ROI and for each subject, normalized by the standard deviations of the blood oxygen level dependent (BOLD) signals. This in vs. out metric was computed as follows:

$$\begin{array}{l} \begin{array}{c} \text{In-field vs}.\\ \text{Out-field Metric} \end{array}=\frac{\text{Preferred }-\text{Nonpreferred}}{\text{Sqrt}\left[\text{std}\left(\text{preferred}\right)\;+\text{std}\left(\text{nonpreferred}\right)\right]} \end{array}$$

Where “preferred” is the BOLD percent signal change evoked by the preferred stimulus for each ROI (i.e., the upper visual field wedge in ventral areas and the lower field wedge in dorsal areas) and “nonpreferred” is the BOLD percent signal change evoked in the ROI by the other, nonpreferred wedge. Standard deviations in BOLD percent signal change were computed within subjects and across runs. The in vs. out metric was contrasted between the CVI and control subject for each visual quadrant ROI as a ratio. A ratio of 100 indicates that the CVI patient and the control demonstrate similar responsivity profiles, while a ratio less than 100 indicates that the representation of the upper/lower visual field is less strongly segregated in the CVI patient than in the control subject.

Both the CVI patient and the control subject were able to maintain adequate central fixation during the fMRI scans allowing for the identification of retinotopically specific cortical activation patterns (CVI patient fixation accuracy: 80%, mean reaction time 640 ms; control subject fixation accuracy: 58%, mean reaction time 729 ms). Functional MRI assessments resulting from the stimulation of horizontal and vertical visual field meridians were used to reveal the boundaries between visual cortical areas and the boundaries of early visual areas (i.e., V1–V3, see Figure 2A). These areas were robustly identified in each hemisphere in both the CVI and control participants suggesting that the overall organization of early visual areas is largely intact in the CVI patient despite the maldevelopment of geniculo-striate projections (compare upper and lower panels of Figure 2B). However, the upper and lower visual field responses showed the most marked difference in the CVI patient mirroring the visual field deficit identified by automated perimetry (described above). Specifically, activation within primary visual cortical representations of the lower visual field (V1d) in the right and left hemisphere of the CVI patient exhibited respectively only 21.5 and 22.2% of the activation observed in the same lower visual field representations in the control participant (Figure 2C). In contrast to those strongly attenuated for the lower visual field, cortical representations of the upper visual field (V1v) exhibited minimal differences in activation between participants (right hemisphere: 91.9% activation of control, left hemisphere: 89.1% activation of control). Again, this pattern is consistent with the inferior visual field deficit characterized by perimetry and the observed reduction in optic radiation tracts along the dorsal branch corresponding to the inferior visual field representation. Finally, in terms of laterality, a greater reduction in activation in the right lower visual field (V1d) compared to the left lover field (V1d) was also observed. This is further consistent with the lateralized visual field deficit in the left side as well as marked reduction in optic radiations observed in the right hemisphere. For comparison, we also examined regional activations in V2 and found a similar pattern to that of V1. Specifically, dorsal areas (lower field representations) exhibited much lower activations in the CVI subject as compared to control (right hemisphere: 33.1% activation of control, left hemisphere: 35.3% activation of control), while the ventral (upper field representations) were similar between CVI and control subjects (right hemisphere: 147.4% activation of control, left hemisphere: 93.7% activation of control). We further noted that the activation ratios between the CVI and control subjects were somewhat smaller in V1 than in V2 (approximately 22% compared to 34%, respectively). This result is consistent with the fact that V2 neurons have larger receptive fields than V1 neurons and thus V2d receives more upper field visual stimulation than does V1d (Zeki, 1978; Smith et al., 2001).

## Discussion

The combination of advanced structural and functional neuroimaging methodologies allows for the characterization of the maldevelopment of visual pathways in relation to assessments of visual function obtained in the clinical setting. In the case presented here, there was a structural-functional correspondence between the clinically observed inferior (and greater on the left) visual field deficit, damage to superior (and greater in the right hemisphere) branches of the optic radiations, and the reduced activation of early visual cortical areas within the inferior visual field (greater in left). This correspondence is in accordance to the known anatomical and functional organization of visual pathways and geniculo-cortical representation of visual field space (Wandell, 1999). To our knowledge, this is the first report combining a multi-modal imaging approach revealing an anatomical and functional correspondence to a visual field deficit in a patient with CVI.

Previous studies have employed various neuroimaging methodologies to investigate the impact of pathology and/or the maldevelopment of the visual system (see Haak et al., 2014; Raz and Levin, 2014 for reviews). For example, Slotnick et al. (2002) reported a case study of a patient with congenital cortical dysgenesis using fMRI retinotopic mapping to characterize a large scale displacement in her cortical visual retinotopic map representation (Slotnick et al., 2002). In another study, Bridge et al. (2009) used a combined structural and diffusion-weighted MRI approach to demonstrate preservation of visual cortex architecture in case of congenital anophthalmia (Bridge et al., 2009). In a unique study by Levin et al. (2010), a combined structural and functional imaging approach was used to characterize white matter tracts and visual cortical maps in an adult individual blinded at the age of three, but who had sight restoration surgery following a corneal and limbal stem-cell transplant procedure in one eye. Even after many years following the surgery, the visual abilities of this individual remained severely limited, and corroborative evidence obtained from combined imaging measurements revealed a number of abnormal visual cortical responses as well as structural (i.e., diffusivity) abnormalities of key whiter matter pathways (Levin et al., 2010).

Regarding CVI, previous studies using diffusion based MRI have identified marked alterations in white matter structure and have further proposed associations between the maldevelopment of key visual pathways and the visual dysfunctions observed in this condition (Ortibus et al., 2011; Bauer et al., 2014; Lennartsson et al., 2014). With regards to the case reported here, a recent review revealed that many individuals with early periventricular damage to the optic radiations (i.e., during third trimester of gestation) often showed normal development of visual field function, perhaps as a result of compensatory neuroplastic reorganization (Guzzetta et al., 2013). These studies suggest that CVI may be associated with a generalized vulnerability in numerous key pathways supporting the developing visual system. However, neuroplastic changes within the developing brain (such as the “re-wiring” of key geniculo-cortical or cortico-cortical connections) may support the sparing of visual function in certain individuals with CVI. Continued studies using advanced multi-modal imaging approaches will likely help in further characterizing these structural-functional associations including the identification of key developmental factors such as the timing, location, and degree of insult.

Apart from the limited observations that can be drawn from a single case study, it is also important to note that the descriptions provided here regarding white matter projections and the degree of functional activation of early visual cortical areas are largely qualitative in nature. Specifically, a reduction in the number of tracts revealed by diffusion-based reconstruction techniques do not necessarily equate to an absolute absence of these connections. Thus, it is important to consider the possibility of uncharacterized reorganization of white matter connections and/or possible false negatives related to the reconstruction process (Johansen-Berg and Behrens, 2006). At the same time, activation measured by fMRI is an indirect measure of brain activity and thus, may not fully characterize underlying physiological and morphological changes. As a result, future studies will require large sample populations in order to fully establish a clear relationship between structural and functional changes in the brain with respect to various outcomes of visual dysfunction.

The combination of structural and functional imaging modalities such as presented here may serve as a key approach in helping to broaden our understanding of brain anatomical-functional relationships as they relate to developmental disorders such as CVI. Observed differences in activation (both in terms of visual areas and between the CVI and control subjects) may also reflect the relative contributions of top-down (i.e., feed-back projections) to both striate and extra-striate cortices from higher order visual areas. At this juncture, it is reasonable to speculate that the optic radiation damage characterized in this CVI subject is responsible for the focal visual field deficit observed. There is evidence however that children with CVI can successfully undergo intensive rehabilitative training and recover a certain degree of visual function (Farrenkopf et al., 1997; McKillop and Dutton, 2008); see also (Poggel et al., 2015). Thus, with functional improvements in overall visual field function, we would suspect changes in the relative activation ratios within these same early visual areas; possibly due to greater top down/feedback influence from higher order areas. To further investigate and confirm this hypothesis, longitudinal scanning, and phase-encoded retinotopic mapping (i.e., to map the retinotopic organization beyond early visual areas) would be needed.

Further, research is needed to fully understand how the developing brain reorganizes itself in relation to sensory and functional recovery and provide a neurological rationale for individually tailored rehabilitative strategies for these patients. Thus, fully characterizing the associations between underlying structural and functional changes with clinical assessments of visual dysfunction may ultimately help us understand how individuals develop, and adapt, in response to early damage to the visual system.

## Author contributions

Designed the study: LM and DS. Collected data: CB and LM. Interpretation and Analysis of data: all authors. Contributed to preparation of the manuscript: all authors.

### Conflict of interest statement

The authors declare that the research was conducted in the absence of any commercial or financial relationships that could be construed as a potential conflict of interest.
